# Comment on “Comparison of the Efficacy of 1064‐ and 730‐nm Picosecond Lasers for Acquired Dermal Melanocytosis”

**DOI:** 10.1111/jocd.70465

**Published:** 2025-10-11

**Authors:** Michelle J. Chang, Justin W. Marson, Alisen Huang

**Affiliations:** ^1^ Department of Dermatology SUNY Downstate Health Sciences University Brooklyn New York USA; ^2^ Department of Dermatology Cedars‐Sinai Medical Center Los Angeles California USA; ^3^ Department of Dermatology Kaiser Permanente Los Angeles Medical Center Los Angeles California USA; ^4^ Dermatology NYC Health and Hospitals – Gotham Brooklyn New York USA

**Keywords:** dermal melanocytosis, laser, picosecond, pigmentation

We are writing in response to the recent publication titled “Comparison of the Efficacy of 1064‐ and 730‐nm Picosecond Lasers for Acquired Dermal Melanocytosis” by Kishi et al. [1]. This study provides valuable insight into the efficacy of treating dermal melanocytosis using a 730‐nm picosecond laser. Patients were either treated with the 730‐nm or 1064‐nm groups, with the 730‐nm group showing higher efficacy and fewer cases of hyperpigmentation.

We would like to present a case of Nevus of Ito split tested with L532‐nm and 1064‐nm picosecond lasers. A 25‐year‐old female with Fitzpatrick skin type III presented with a congenital gray‐blue patch involving the right upper back extending to the chest and lateral neck. The lesion was biopsy‐proven Nevus of Ito and had significantly enlarged since birth, growing with the patient to cover the anterior shoulder and reportedly darkening in color. A test spot previously performed at an outside clinic with a picosecond laser showed some improvement in pigmentation; however, the patient moved to the United States shortly afterward. She presented to our clinic requesting further treatment of the lesion.

To compare the efficacy between laser wavelengths, the anterior shoulder was divided into two sections and treated with one pass of either a 532‐nm (fluence 2.0 J/cm^2^, spot size 3 mm) or 1064‐nm picosecond laser (fluence of 2.5 J/cm^2^, spot size 3 mm) with an endpoint of immediate whitening. After 4 weeks, the 532‐nm treated area showed greater lightening in pigmentation as subjectively graded by both the clinician and the patient, and the patient expressed higher satisfaction in this test section (Figure 1). The remainder of the shoulder was treated with 532 nm, yielding continued improvement in pigmentation (Figure 2).

**FIGURE 1:**
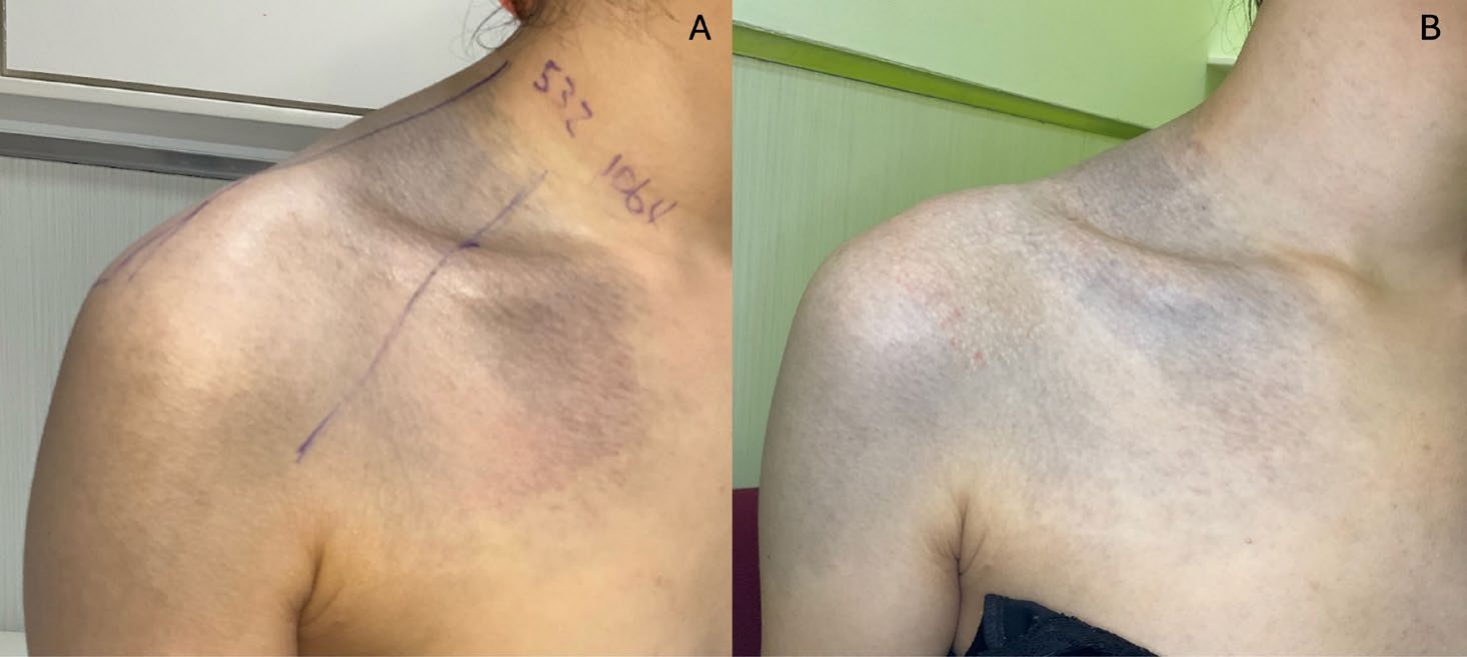
(A) Map of test spots 532‐nm and 1064‐nm. (B) Four weeks later, 532‐nm showed greater improvement compared to 1064‐nm.

**FIGURE 2:**
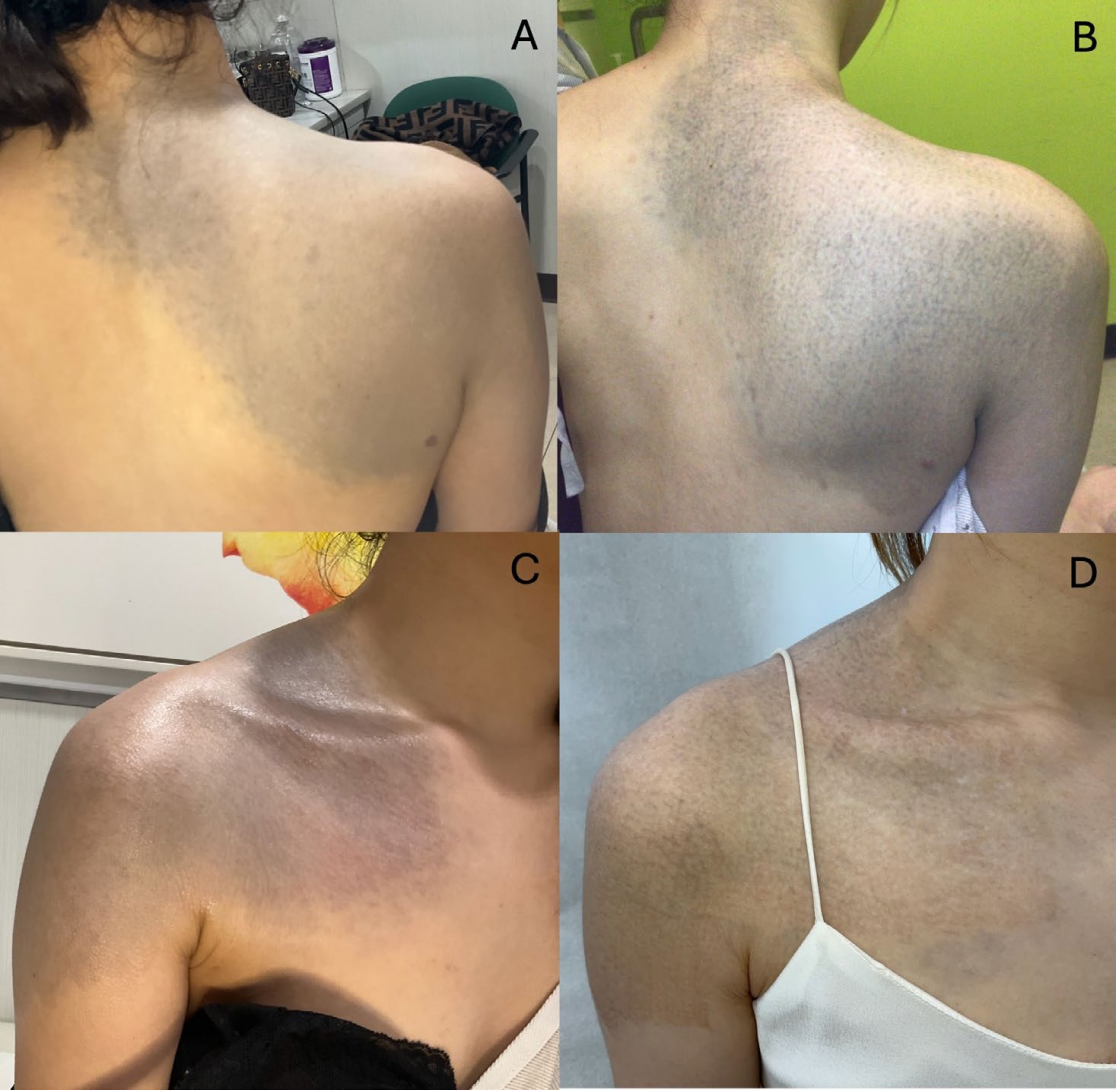
(A, B) Before and after of the back and posterior shoulder after four treatments. (C, D) Before and after of the chest and anterior shoulder after four treatments over 1 year.

Dermal melanocytosis can be challenging to treat in patients with skin of color, as energy‐based laser devices utilize photothermolysis to selectively induce thermal damage, in this case, to the chromophore melanin. Due to the increased melanin content in these patients, they are at a higher risk of pigmentary‐associated adverse events such as hyperpigmentation. The 532‐, 730‐, and 1064‐nm picosecond lasers have all been shown to be safe in this patient population [1, 2, 3]. Additionally, the short pulse width in picosecond lasers allows for rapid disruption of melanosomes while minimizing damage to surrounding structures [4]. Both 532 nm and 730 nm are more strongly absorbed by melanin than 1064 nm. Although 1064 nm and 730 nm penetrate deeper into the dermis, the 532‐nm wavelength demonstrates greater fragmentation of melanin [5, 6]. Therefore, in addition to the 730‐nm picosecond laser, we propose the 532‐nm laser with a short pulse width as another potential treatment option for dermal melanocytosis. Future clinical studies are necessary to compare the efficacy of this wavelength with that of 730‐nm laser and 1064‐nm laser.

## Author Contributions

Dr. Alisen Huang managed patient care. Dr. Michelle J. Chang drafted the initial manuscript. All authors contributed to the revision process and approved the final version of the manuscript.

## Ethics Statement

Informed consent was obtained from the patient for publication of this case and any accompanying images.

## Conflicts of Interest

The authors declare no conflicts of interest.

## Linked Articles

Comparison of the Efficacy of 1064‐ and 730‐nm Picosecond Lasers for Acquired Dermal Melanocytosis. https://doi.org/10.1111/jocd.70123

## Data Availability

Data sharing not applicable to this article as no datasets were generated or analyzed during the current study.
